# Prevalence of body composition phenotypes and their associations with glycemic, lipidic, and inflammatory biomarkers: a population-based study

**DOI:** 10.1590/0102-311XEN109823

**Published:** 2024-06-14

**Authors:** Giovanna Mozzaquattro Nascimento, Giana Zarbato Longo, Aline Valmorbida, Fabrícia Geralda Ferreira, Erasmo Benicio Santos de Moraes Trindade

**Affiliations:** 1 Universidade Federal de Santa Catarina, Florianópolis, Brasil.; 2 Universidade Federal de Viçosa, Viçosa, Brasil.

**Keywords:** Obesity, Sarcopenia, Inflammation, Insulin Resistance, Phenotype, Obesidade, Sarcopenia, Inflamação, Resistência à Insulina, Fenótipo, Obesidad, Sarcopenia, Inflamación, Resistencia a la Insulina, Fenotipo

## Abstract

We aimed to verify the prevalence of body composition phenotypes and the association of glycemic, lipidic, and inflammatory biomarkers with such phenotypes. This is a cross-sectional, population-based study, with 720 participants aged 20 to 59 years. Body composition was assessed by dual-energy X-ray absorptiometry. Obesity was defined as body fat percentage ≥ 25% in males and ≥ 32% in females and sarcopenia by appendicular muscle mass index < 7.0kg/m^2^ in males and < 5.5kg/m^2^ in females. Sarcopenic obesity (SO) was defined as the presence of both sarcopenia and obesity. The prevalence of obesity, sarcopenia, and SO were 62.5%, 4.5%, and 6.2%, respectively. The association between biomarkers and phenotypes was verified using multinomial logistic regression models adjusted for confounding factors. The models showed that increased glycemia (OR = 3.39; 95%CI: 1.83-6.27), total cholesterol (TC) (OR = 2.24; 95%CI: 1.35-3.70), LDL-c (OR = 1.01; 95%CI: 1.00-1.02), VLDL-c (OR = 1.04; 95%CI: 1.02-1.06), non-HDL-c (OR = 1.02; 95%CI: 1.01-1.03), triglycerides (Tg) (OR = 3.66; 95%CI: 2.20-6.06), and decreased HDL-c (OR = 0.97; 95%CI: 0.95-0.98) were significantly associated with the obesity phenotype. Increased HOMA-IR (OR = 3.94; 95%CI: 1.69-9.21), LDL-c (OR = 1.01; 95%CI: 1.00-1.02), non-HDL-c (OR = 1.01; 95%CI: 1.00-1.02), and hs-CRP (OR = 2.42; 95%CI: 1.04-5.66) were independently associated with SO phenotype. Our findings indicate that increased glycemia, TC, Tg, LDL-c, VLDL-c, non-HDL-c, and decreased HDL-c may be indicators of the obesity phenotype and that increased hs-CRP, HOMA-IR, LDL-c, and non-HDL-c appear to be indicators of the SO phenotype. Those parameters may be used as additional markers for screening.

## Resumo

Objetivou-se verificar a prevalência de fenótipos corporais e suas associações com biomarcadores dos perfis glicídico, lipídico e inflamatório. Trata-se de um estudo transversal, de base populacional, com 720 indivíduos de 20 a 59 anos. A composição corporal foi avaliada por absorciometria com raios X de dupla energia. Obesidade foi definida como percentual de gordura corporal ≥ 25% em homens e ≥ 32% em mulheres e sarcopenia pelo índice de massa muscular apendicular < 7,0kg/m^2^ em homens e < 5,5kg/m^2^ em mulheres. A obesidade sarcopênica foi definida como a coexistência de sarcopenia e obesidade. As prevalências de obesidade, sarcopenia e obesidade sarcopênica foram de 62,5%, 4,5% e 6,2%, respectivamente. A associação entre biomarcadores e fenótipos foi verificada por meio de modelos de regressão logística multinomial ajustados por variáveis de confusão. Os modelos mostraram que níveis aumentados de glicemia (OR = 3,39; IC95%: 1,83-6,27), colesterol total (OR = 2,24; IC95%: 1,35-3,70), LDL-c (OR = 1,01; IC95%: 1,00-1,02), VLDL-c (OR = 1,04; IC95%: 1,02-1,06), não HDL-c (OR = 1,02; IC95%: 1,01-1,03), triglicerídeos (OR = 3,66; IC95%: 2,20-6,06) e diminuição do HDL-c (OR = 0,97; IC95%: 0,95-0,98) foram significativamente associados ao fenótipo de obesidade. Índices aumentados de HOMA-IR (OR = 3,94; IC95%: 1,69-9,21), LDL-c (OR = 1,01; IC95%: 1,00-1,02), não HDL-c (OR = 1,01; IC95%: 1,00-1,02) e PCR-us (OR = 2,42; IC95%: 1,04-5,66) foram independentemente associados ao fenótipo de obesidade sarcopênica. Nossos resultados sugerem que níveis aumentados de glicemia, colesterol total, triglicerídeos, LDL-c, VLDL-c, não HDL-c e graus reduzidos de HDL-c são indicadores do fenótipo de obesidade e que o aumento em níveis de PCR-us, HOMA-IR, LDL-c e não HDL-c são indicadores do fenótipo de obesidade sarcopênica. Esses parâmetros podem ser usados como marcadores adicionais para triagem.

## Resumen

El objetivo de este estudio fue evaluar la prevalencia de fenotipos corporales y sus asociaciones con biomarcadores de perfiles lipídicos, glucídicos e inflamatorios. Se trata de un estudio transversal, de base poblacional, realizado con 720 individuos de entre 20 y 59 años. La composición corporal se evaluó mediante absorciometría de rayos X de energía dual. La obesidad se estimó como porcentaje de grasa corporal ≥ 25% en hombres y ≥ 32% en mujeres, y la sarcopenia como índice de masa muscular apendicular < 7,0kg/m^2^ en hombres y < 5,5kg/m^2^ en mujeres. La obesidad sarcopénica se evaluó como la coexistencia de sarcopenia y obesidad. Las prevalencias de obesidad, sarcopenia y obesidad sarcopénica fueron del 62,5%, 4,5% y 6,2%, respectivamente. La asociación entre biomarcadores y fenotipos se comprobó mediante modelos de regresión logística multinomial ajustados por variables de confusión. Los modelos mostraron que el incremento de los niveles de glucosa en la sangre (OR = 3,39; IC95%: 1,83-6,27), colesterol total (OR = 2,24; IC95%: 1,35-3,70), LDL-c (OR = 1,01; IC95%: 1,00-1,02), VLDL-c (OR = 1,04; IC95%: 1,02-1,06), no HDL-c (OR = 1,02; IC95%: 1,01-1,03), triglicéridos (OR = 3,66; IC95%: 2,20-6,06) y disminución de HDL-c (OR = 0,97; IC95%: 0,95-0,98) se asociaron significativamente con el fenotipo de obesidad. Las tasas aumentadas de HOMA-IR (OR = 3,94; IC95%: 1,69-9,21), LDL-c (OR = 1,01; IC95%: 1,00-1,02), no-HDL-c (OR = 1,01; IC95%: 1,00-1,02) y PCR-us (OR = 2,42; IC95%: 1,04-5,66) se asociaron de manera independiente con el fenotipo de obesidad sarcopénica. Los resultados demuestran que el aumento de los niveles de glucosa en la sangre, colesterol total, triglicéridos, LDL-c, VLDL-c, no-HDL-c y grados reducidos de HDL-c son indicadores del fenotipo de obesidad y que el incremento de los niveles de PCR-us, HOMA-IR, LDL-c y no-HDL-c son indicadores del fenotipo de obesidad sarcopénica. Estos parámetros se pueden utilizar como marcadores adicionales para el cribado.

## Introduction

Body composition phenotypes are determined by changes in body composition that may or may not be related to changes in biomarkers and the presence of diseases ^1^. Both phenotypes of obesity and sarcopenia have been associated, in isolation, with a chronic, low-grade inflammatory state and, in addition, they can cause or accentuate cases of insulin resistance (IR) and dysregulation in lipidic metabolism. When IR is prolonged, it results in the exacerbation of the inflammatory state, which in turn is associated with muscle catabolism, demonstrating a cyclical relationship. It is well established that excess body fat and low muscle mass are isolated and directly associated with dyslipidemia, and considered predisposing factors ^2^. Furthermore, some biomarkers such as c-reactive protein (CRP) ^3^ and blood glucose ^4^ have already been associated with sarcopenic obesity (SO) in older individuals. Although the association between these biomarkers and SO is usually considered bilateral, increased levels of IR can predict the chance of individuals presenting SO in older adults ^5^.

SO is considered a novel body composition phenotype that is characterized by reduced muscle mass coexisting with increased fat mass. Due to the strong interconnection between adipose tissue and skeletal muscle tissue, when sarcopenia occurs simultaneously with obesity, it results in a double metabolic burden and increased risks of adverse health complications, such as increased risk of developing cardiovascular, endocrine, and metabolic disorders ^6^. The recommendation for definition of the sarcopenia phenotype is the coexistence of low muscle strength and low muscle quantity or quality ^7^. Despite being a recommendation from the European Working Group on Sarcopenia in Older People 2 (EWGSOP2) ^7^, a recent systematic review of definitions and diagnostic criteria for SO identified that none of the included studies considered sarcopenia according to a coexistence of reduced muscle strength and quantity. In most studies, the diagnostic of SO was based on the coexistence of obesity and sarcopenia (defined as low or reduced skeletal muscle mass) ^8^.

Furthermore, the estimated worldwide prevalence of SO is 5-10% in adults of both sexes. These numbers were shown to increase directly proportional to age, reaching its peak of, approximately, 50% in individuals aged 80 years or more ^7^. However, despite the higher prevalence of SO in older populations and sarcopenia having long been associated with aging, it is now recognized that the development of sarcopenia begins earlier in life ^7^, which allows for early diagnosis of sarcopenia phenotype in younger individuals. Despite these facts, the studies carried out on SO to date have included only aged individuals and older adults in their sample, with a mean age of 64.8 ± 4.5 years being identified on a systematic review of definitions and diagnostic criteria for SO ^8^. Therefore, studies on sarcopenia and SO phenotypes in younger adults are lacking, which in turn hinders the identification of biomarkers that could aid to screen this population.

Although some studies already report the prevalence of the SO phenotype, to the best of our knowledge, no previous study has observed the prevalence of body composition phenotypes (obesity, sarcopenia, and SO) in Brazilian adults and young adults. Nor did they simultaneously verify the association between IR, lipid profile, and inflammation and phenotypes. Therefore, this study aimed to estimate the prevalence of body composition phenotypes: obesity, sarcopenia, and SO, as well as the associations of glycemic, lipidic, and inflammatory biomarkers with these phenotypes of body composition in Brazilian adults and young adults.

## Material and methods

### Study design and participants

This study included data derived from a cross-sectional, population-based study, which aimed to evaluate the health condition of the adult population in a city in Minas Gerais State, Brazil (2012-2014). The research was conducted with adult individuals aged 20 to 59 years, of both sexes, who lived in the state’s urban area, regardless of the health status. To ensure that the sample was representative of the municipality’s population, census data regarding sex, age, and years of education, as well as the necessary sample size, were considered. Exclusion factors were pregnant women, bedridden individuals, amputees, and those unable to undergo anthropometric or body composition measurements, or to respond to the questionnaire. Individuals with chronic kidney disease were not included in this study sample. More details about study procedures are described elsewhere ^9^.

The sampling process was probabilistic, without replacement, using a 2-stage conglomerate sampling process (census and domicile). This study is reported following the Strengthening the Reporting of Observational Studies in Epidemiology (STROBE) statement ^10^. The sample size was calculated in the public domain Open Epi application, online version 3.03a (http://www.OpenEpi.com), considering the following parameters: estimated population of 43,431 individuals ^11^, 95% confidence level, expected prevalence of 50%, 4.5% predicted sample error, and 1.4 design effect. The calculated sample was added with 10% to control for confounding factors. The required sample size was estimated to be 722 individuals.

### Measurements

#### Characterization parameters

A structured questionnaire was applied by trained researchers via face-to-face interviews at each participants household to collect data on sex, age range, years of education, self-reported skin color, marital status, socioeconomic level according to the Brazilian Economic Classification Criteria (CCEB) ^12^, smoking status, presence or absence of diseases, physical activity, and sedentary behavior (screen time ≥ 4 hours/day) ^13^.

The *International Physical Activity Questionnaire* (IPAQ), validated for the Brazilian population ^14^, was used to quantify the time spent in leisure-time physical activities over a week. Individuals who obtained a result ≥ 150 minutes were classified as physically active and those with a result < 150 minutes were classified as insufficiently active.

#### Nutritional status and body composition

Weight and height were measured following standard methods ^15^. A Welmy stadiometer (https://www.welmy.com.br/) and a Tanita digital scale (https://www.tanita.com/) were used to measure height and weight, respectively. The body mass index (BMI) was calculated by the ratio between body mass (kg) and height (m) squared. Percentage of body fat and appendicular muscle mass (kg) were determined by dual-energy X-ray absorptiometry (DXA) (Lunar DPX, General Electric; https://www.gehealthcare.com.br) using an analysis software (Lunar enCORE; https://www.gehealthcare.com/products/bone-and-metabolic-health/encore-software-platform). All DXA assessments were performed by the same specialized technician and the equipment was calibrated daily using the standard procedure of the enCORE user’s manual. The appendicular muscle mass index was obtained by the sum of the muscle mass of the arms and legs divided by the height squared ^16^.

#### Definition of body composition phenotypes

Obesity was defined as a high percentage of body fat for sex, considering the following cutoff points: percentage of body fat ≥ 25% for males and ≥ 32% for females ^17^ (measured by DXA). Sarcopenia was defined by the appendicular muscle mass index < 7.0kg/m^2^ in males and < 5.5kg/m^2^ in females ^18^. These cutoff points were recommended by the EWGSOP2 ^7^. SO was defined as the coexistence of obesity and sarcopenia. Individuals were classified into four phenotypes: without sarcopenia and obesity, obesity only, sarcopenia only, and SO.

#### Biochemical parameters

Blood collection was performed with individuals fasting for 12 hours. It was performed via peripheral intravenous puncture using the Vacutainer vacuum system (Becton Dikinson; https://www.bd.com). Fasting blood glucose was determined by the enzymatic glucose-oxidase method and classified as increased when ≥ 100mg/dL ^19^. Fasting insulin was determined by ELISA method using the human insulin kit (Human Insulin ELISA Kit, Linco Research; https://lincoresearch.com/). IR was determined by homeostatic model assessment (HOMA-IR) ^20^ index when HOMA-IR ≥ 2.71 ^21^. Total cholesterol (TC), very low-density lipoprotein (VLDL-c), high density lipoprotein (HDL-c), and triglycerides (Tg) were measured by the enzymatic colorimetric method. TC and Tg were classified as increased when ≥ 190mg/dL and ≥ 150mg/dL, respectively ^22^. Low density lipoprotein (LDL-c) was calculated using Friedewald formula ^23^. Non-HDL-c was calculated by TC minus HDL-c. Ultrasensitive c-reactive protein (hs-CRP) was quantified by immunoturbidimetric testing, using the hs-CRP K079 kit (Bioclin; https://www.bioclin.com.br/) at a 0.0313mg/dL sensitivity and classified as increased when ≥ 1mg/L ^20^.

### Statistical analysis

Data were entered in duplicate using the Epidata software (http://www.epidata.dk/) and checked by the “data compare” module. Statistical analyses were conducted in Stata version 13.0 (https://www.stata.com) using the *svy* command set for weighting, according to distribution by sex, age group, and years of education. The set of *svy* commands considered the study complex sample design ^24^.

Data normality was verified by asymmetry coefficient, graphical representation, and Shapiro-Wilk test. Data description was performed using mean, standard error (SE), medians, interquartile intervals, and their respective 95% confidence intervals (95%CI). Categorical variables were presented as proportion and 95%CI.

Multinomial logistic regression models were developed to identify the association of biomarkers (independent variables) and body composition phenotypes (dependent variable), presented in estimated odds ratio (OR) and 95%CI. The phenotype without SO was used as reference for the dependent variable. The model was adjusted for confounding variables, which were determined by biological and epidemiological relevance (sex, age group, years of education, and level of physical activity). Values of p < 0.05 and 95%CI without overlapping were considered statistically significant.

## Results

In total, 1,257 individuals were interviewed. Of the total number of interviewees, 308 did not complete the laboratory tests and 229 did not undergo DXA; the final sample was 720 individuals. Females constituted 50.1% of the total. The age group with the highest frequency was 30-39 (27%), followed by 50-59 (26.9%). Most participants had 12 or more years of education, being the highest 78.2% in the sarcopenia only group, followed by 68.6% in the SO. More than half of the individuals were non-white in all phenotype groups, with significant difference on the obesity only group, in which non-whites constituted 65.8% (95%CI: 58.2-72.7) and whites 34.2% (95%CI: 27.3-41.8). Most individuals were classified as medium socioeconomic level (C), nonsmoker, without diabetes and hypertension, physically inactive, and with sedentary behavior in all groups. The sedentary behavior was significantly higher in the sarcopenia only group when compared to the nonsedentary in the same group. Table 1 presents these data.

**Table 1:** Characterization of the study sample according to sociodemographic and behavioral variables and presence of diseases. Viçosa, Minas Gerais State, Brazil, 2012-2014.

Characteristics	Without sarcopenia and obesity	Obesity only	Sarcopenia only	Sarcopenic obesity
	% (95%CI)	% (95%CI)	% (95%CI)	% (95%CI)
Sex				
Female	28.1 (20.7-36.9)	56.5 (49.9-62.9)	50.7 (32.4-68.9)	80.1 (65.5-89.5)
Male	71.9 (63.1-79.3)	43.5 (37.1-50.1)	49.3 (31.1-67.6)	19.9 (10.5-34.5)
Age group (years)				
20-29	35.9 (24.8-48.7)	13.2 (8.6-19.7)	54.6 (38.1-70.1)	27.9 (15.8-44.4)
30-39	28.4 (20.7-37.6)	25.3 (19.6-32.0)	27.8 (14.8-46.1)	37.0 (23.8-52.6)
40-49	14.6 (9.5-21.9)	29.7 (23.5-36.8)	15.0 (6.3-31.6)	14.3 (5.6-32.1)
50-59	21.0 (13.0-32.1)	31.8 (26.5-37.7)	2.6 (0.4-1.7)	20.7 (11.8-33.7)
Education (years)				
1-4	15.6 (7.9-28.6)	25.0 (16.4-36.3)	2.8 (0.3-20.0)	0.0 (0.0-0.0)
5-8	14.8 (9.1-23.1)	19.4 (14.3-25.7)	0.0 (0.0-0.0)	2.2 (0.03-15.7)
9-11	24.2 (18.4-31.2)	19.2 (15.2-24.0)	19.0 (9.0-35.7)	29.2 (17.6-44.3)
≥ 12	45.4 (30.8-60.8)	36.4 (24.9-49.7)	78.2 (61.3-89.1)	68.6 (52.1-81.4)
Skin color				
White	46.2 (35.7-57.0)	34.2 (27.3-41.8)	49.8 (32.7-66.9)	47.3 (31.8-63.4)
Non-white	53.8 (43.0-64.3)	65.8 (58.2-72.7)	50.2 (33.1-67.3)	52.7 (36.6-68.2)
Marital status				
Single/Divorced/Widowed	55.5 (43.0-67.3)	35.8 (26.7-46.0)	68.1 (49.3-82.4)	50.8 (38.0-63.5)
Married	44.5 (32.7-57.0)	64.2 (54.0-73.3)	31.9 (17.6-50.7)	49.2 (36.5-62.0)
Socioeconomic level				
High (A e B)	26.2 (18.0-36.4)	26.8 (19.5-35.6)	24.4 (13.0-40.9)	18.7 (10.8-30.4)
Medium (C)	66.4 (56.2-75.3)	64.5 (57.2-71.1)	63.3 (45.1-78.3)	74.0 (62.8-82.8)
Low (D e E)	7.4 (3.2-16.3)	8.7 (3.2-16.3)	12.4 (3.4-36.5)	7.2 (2.2-21.2)
Smoking status				
Nonsmoker	70.8 (59.8-79.8)	59.1 (52.2-65.7)	85.8 (67.5-94.6)	86.2 (71.9-93.8)
Ex-smoker	11.5 (6.9-18.5)	29.7 (22.2-38.6)	3.2 (0.4-21.7)	5.4 (1.7-16.0)
Smoker	17.7 (11.6-26.0)	11.1 (7.9-15.3)	11.0 (3.5-29.6)	8.4 (3.3-19.6)
Diabetes				
Yes	2.0 (0.7-5.5)	8.9 (5.0-15.2)	2.6 (0.4-16.9)	2.1 (0.3-15.7)
No	98.0 (94.5-99.3)	91.1 (84.8-95.0)	97.4 (83.1-99.6)	97.9 (84.3-99.7)
Hypertension				
Yes	28.2 (19.9-38.3)	45.2 (39.3-51.2)	20.5 (8.6-41.3)	17.3 (7.5-35.0)
No	71.8 (61.7-80.1)	54.8 (48.7-60.7)	79.5 (58.6-91.3)	82.7 (65.0-92.5)
Physical activity level (minutes)				
< 150	70.7 (58.1-80.7)	81.0 (73.0-87.0)	80.6 (63.0-91.1)	81.8 (68.8-90.2)
≥ 150	29.3 (19.2-41.9)	19.0 (13.0-27.0)	19.4 (8.9-37.0)	18.2 (9.8-31.2)
Sedentary behavior				
Yes (≥ 4 hours)	54.3 (40.6-67.4)	50.1 (42.6-57.7)	85.4 (64.0-95.1)	58.7 (44.6-71.5)
No (< 4 hours)	45.7 (32.6-59.4)	49.9 (42.3-57.4)	14.6 (4.9-36.0)	41.3 (28.5-55.3)

95%CI: 95% confidence interval.

The prevalence of sarcopenia and SO in the population were, respectively, 4.52% (95%CI: 3.09-6.58) and 6.17% (95%CI: 4.36-8.66). The prevalence of SO was greater in females (9.87%; 95%CI: 7.04-13.67) than in males (2.46%; 95%CI: 1.11-5.38). An important characteristic in this sample is the high prevalence of obesity (62.48%; 95%CI: 56.37-68.22), which was also more prevalent in females (70.5%; 95%CI: 62.86-77.15) than in males (54.43; 95%CI: 46.25-62.37) (Table 2).

**Table 2:** Prevalence of body composition phenotypes according to sex. Viçosa, Minas Gerais State, Brazil, 2012-2014.

Body composition phenotype	Males	Females	Total
% (95%CI)	% (95%CI)	% (95%CI)
Without sarcopenia and obesity	38.64 (31.18-46.47)	15.05 (11.12-20.06)	26.82 (22.74-31.34)
Obesity only	54.43 46.25-62.37)	70.50 (62.86-77.15)	62.48 (56.37-68.22)
Sarcopenia only	4.47 (2.37-8.27)	4.58 (2.97-7.00)	4.52 (3.09-6.58)
Sarcopenic obesity	2.46 (1.11-5.38)	9.87 (7.04-13.67)	6.17 (4.36-8.66)

95%CI: 95% confidence interval.

Table 3 describes the biochemical variables, body composition, and nutritional status of the sample according to body composition phenotypes. We observed higher means for TC (≥ 190mg/dL), HOMA-IR (≥ 2.71), and hs-CRP (≥ 1mg/L) in individuals with the obesity and SO phenotypes. The highest means of BMI were in individuals diagnosed with obesity (according to DXA), and the lowest means of BMI in individuals with sarcopenia, followed by those with SO. We highlight that the group with SO had mean BMI values compatible with a diagnosis of normal weight.

**Table 3:** Distribution of nutritional status, body composition, and biochemical variables according to body composition phenotypes in adults. Viçosa, Minas Gerais State, Brazil, 2012-2014 (n = 720).

Variables	Without sarcopenia and obesity		Obesity	
	Mean ± SE or Median (IQR)	95%CI	Mean ± SE or Median (IQR)	95%CI
BMI (kg/m^2^)	22.46 ± 0.22 ^a^	22.01-22.90	27.85 ± 0.25 ^a,b,c^	27.33-28.37
Body fat percentage (%)	20.31 ± 0.75 ^a,c^	18.76-21.85	36.51 ± 0.42 ^a,b^	35.65-37.37
Muscle mass (kg)	50.39 ± 0.71 ^a^	48.93-51.85	45.51 ± 0.53 ^a^	44.41-46.60
Appendicular muscle mass index (kg/m^2^)	7.68 ± 0.09 ^a^	7.50-7.86	7.45 ± 0.07 ^b^	7.29-7.60
Fasting blood glucose (mg/dL)	82.00 (76.00-87.00) ^a^	80.53-83.47	85.00 (78.00-94.00) ^a^	83.53-86.47
HOMA-IR (units)	1.03 (0.71-1.60) ^a^	0.89-1.18	1.84 (1.23-2.76) ^a,b^	1.71-1.97
TC (mg/dL)	178.15 ± 3.01 ^a^	171.98-184.31	199.24 ± 2.35 ^a,b^	194.42-204.05
VLDL-c (mg/dL)	17.40 (13.80-24.00) ^a^	16.22-18.58	24.80 (16.40-36,00) ^a,b^	21.57-28.03
HDL-c (mg/dL)	50.06 ± 1.09	47.81-52.30	46.92 ± 1.19 ^a^	44.48-49.35
Tg (mg/dL)	87.00 (69.00-120.00) ^a^	81.12-92.88	124.00 (82.00-180.00) ^a,b^	107.83-140.17
LDL-c (mg/dL)	107.49 ± 2.92 ^a^	101.51-113.47	121.20 ± 1.55 ^a,b^	118.03-124.37
Non-HDL-c (mg/dL)	128.09 ± 3.18 ^a^	121.58-134.61	152.32 ± 2.02 ^a,b^	148.19-156.45
hs-CRP (mg/L)	0.67 (0.26-1.59) ^a^	0.49-0.85	1.48 (0.68-3.15) ^a,b^	1.20-1.76
Variables	Sarcopenia		Sarcopenic obesity	
	Mean ± SE or Median (IQR)	95%CI	Mean ± SE or Median (IQR)	95%CI
BMI (kg/m^2^)	19.12 ± 0.27 ^a,b,c^	18.57-19.67	22.00 ± 0.27 ^c^	21.44-22.56
Body fat percentage (%)	23.25 ± 1.39 ^b,c^	20.39-26.09	37.02 ± 0.52 ^c^	35.94-38.10
Muscle mass (kg)	40.31 ± 1.35 ^a^	37.55-43.07	34.27 ± 0.72 ^a^	32.80-35.75
Appendicular muscle mass index (kg/m^2^)	5.99 ± 0.14 ^a,b,c^	5.70-6.28	5.41 ± 0.08 ^a,b,c^	5.25-5.57
Fasting blood glucose (mg/dL)	81.00 (77.00-86.00)	78.06-83.94	84.00 (77.00-90.00)	80.08-87.92
HOMA-IR (units)	0.83 (0.63-1.22) ^b^	0.59-1.07	1.39 (1.02-2.34)	0.94-1.83
TC (mg/dL)	167.71 ± 5.16 ^b,c^	157.14-178.27	194.98 ± 6.78 ^c^	181.09-208.86
VLDL-c (mg/dL)	13.80 (11.20-17.40) ^a,b^	12.43-15.17	19.20 (14.80-27.00) ^a^	16.95-21.45
HDL-c (mg/dL)	52.45 ± 2.03	48.28-56.61	53.97 ± 1.70 ^a^	50.49-57.46
Tg (mg/dL)	69.00 (56.00-87.00) ^a,b^	62.14-75.86	96.00 (74.00-135.00) ^b^	84.73-107.27
LDL-c (mg/dL)	99.08 ± 4.91 ^b^	89.03-109.14	119.58 ± 5.73	107.83-131.32
Non-HDL-c (mg/dL)	115.26 ± 5.15 ^b,c^	104.70-125.82	141.00 ± 6.69 ^c^	127.29-154.72
hs-CRP (mg/L)	0.56 (0.21-1.52) ^b^	0.02-1.09	1.32 (0.70-4.02)	0.59-2.05

95%CI: 95% confidence interval; BMI: body mass index; HDL-c: high-density lipoprotein; HOMA-IR: homeostatic model assessment - insulin resistance; hs-CRP: ultrasensitive c-reactive protein; IQR: interquartile range; LDL-c: low-density lipoprotein; SE: standard error; TC: total cholesterol; Tg: triglycerides; VLDL-c: very high-density lipoprotein.

Note: equal letters show statistically significant difference in the mean between the groups, according to the 95%CI.

Table 4 presents the results of the multinomial logistic regression models adjusted for sex, age, years of education, and level of physical activity. It was observed that individuals with increased blood glucose (≥ 100mg/dL) had 239% greater odds of being obese and 482% greater odds of having sarcopenia compared to those with normal blood glucose (< 100mg/dL). Individuals with increased HOMA-IR (≥ 2.71) had 430% greater odds of being obese and 294% greater odds of having SO compared to those with normal HOMA-IR (< 2.71). As for lipidic biomarkers, it was found that individuals with increased TC (≥ 190mg/dL) had 124% greater odds of being obese compared to those with normal levels of TC (< 190mg/dL). In addition, an increase of 1mg/dL of LDL-c and VLDL-c was associated with greater odds of the individual being obese, with the values OR = 1.01 (95%CI: 1.00-1.02) and OR = 1.04 (95%CI: 1.02-1.06), respectively. The increase in HDL-c presented an inverse association with the chance of the individual being obese, with an increase of 1mg/dL of HDL-c reducing the odds by 3% (OR = 0.97; 95%CI: 0.95-0.98). Participants with increased Tg (> 150mg/dL) showed 266% greater odds in the chance of being obese compared to those with normal levels of Tg (≤ 150mg/dL). Regarding inflammatory biomarker, it was found that high hs-CRP (≥ 1mg/L) was associated with 152% greater odds of the individual being obese and 142% of having SO compared to those with lower hs-CRP (< 1mg/L). All associations held statistical significance (p < 0.05), as shown in Table 4.

**Table 4:** Final multinomial logistic regression models for factors associated with body composition phenotypes. Viçosa, Minas Gerais State, Brazil, 2012-2014.

Variables	Obesity only		Sarcopenia only		Sarcopenic obesity	
	OR (95%CI)	p-value	OR (95%CI)	P-value	OR (95%CI)	p-value
High fasting blood glucose (≥ 100mg/dL) *	3.62 (2.23-5.91)	2.03	2.07 (0.44-9.68)	0.16	0.65 (0.12-3.52)	0.23
High fasting blood glucose (≥ 100mg/dL) **	3.39 (1.83-6.27)	< 0.01 ***	5.82 (1.31-25.73)	0.022 *	1.42 (0.20-10.09)	0.72
High HOMA-IR (≥ 2.71) *	5.23 (3.06-8.96)	1.72	1.29 (0.42-3.91)	0.16	3.30 (1.50-7.24)	0.19
High HOMA-IR (≥ 2.71) **	5.30 (3.06-9.18)	< 0.01 ***	1.47 (0.49-4.48)	0.48	3.94 (1.69-9.21)	0.003 ***
High TC (≥ 190mg/dL) *	2.83 (1.83-4.39)	1.45	0.64 (0.30-1.36)	0.19	1.96 (1.04-3.71)	0.17
High TC (≥ 190mg/dL) **	2.24 (1.35-3.70)	0.003 ***	0.79 (0.35-1.77)	0.55	1.65 (0.78-3.52)	0.18
VLDL-c (mg/dL) *	1.04 (1.02-1.06)	0.80	0.94 (0.88-1.01)	0.50	1.01 (0.98-1.03)	0.20
VLDL-c (mg/dL) **	1.04 (1.02-1.06)	< 0.01 ***	0.94 (0.87-1.02)	0.16	1.01 (0.98-1.04)	0.46
HDL-c (mg/dL) *	0.99 (0.97-1.00)	4.73	1.00 (0.99-1.03)	0.10	1.02 (1.00-1.03)	0.10
HDL-c (mg/dL) **	0.97 (0.95-0.98)	< 0.01 ***	0.99 (0.97-1.02)	0.74	0.98 (0.97-1.00)	0.12
High Tg (≥ 150mg/dL) *	3.76 (2.34-6.04)	1.68	0.67 (0.20-2.26)	0.18	1.60 (0.76-3.39)	0.21
High Tg (≥ 150mg/dL) **	3.66 (2.20-6.06)	< 0.01 ***	0.94 (0.30-2.98)	0.92	2.01 (0.98-4.14)	0.058
LDL-c (mg/dL) *	1.01 (1.00-1.02)	0.54	0.99 (0.98-1.00)	0.44	1.01 (1.00-1.02)	0.62
LDL-c (mg/dL) **	1.01 (1.00-1.02)	0.027 ***	1.00 (0.98-1.01)	0.678	1.01 (1.00-1.02)	0.018 ***
Non HDL-c (mg/dL) *	1.02 (1.01-1.03)	0.18	0.99 (0.98-1.00)	0.72	1.01 (1.00-1.02)	0.06
Non HDL-c (mg/dL) **	1.02 (1.01-1.03)	< 0.01 ***	0.99 (0.98-1.00)	0.452	1.01 (1.00-1.02)	0.008 ***
High hs-CRP (≥ 1mg/L) *	3.28 (2.33-4.60)	1.31	1.57 (0.62-3.98)	0.14	3.04 (1.40-6.63)	0.14
High hs-CRP (≥ 1mg/L) **	2.52 (1.80-3.54)	< 0.01 ***	1.53 (0.60-3.88)	0.359	2.42 (1.04-5.66)	0.041 ***

95%CI: 95% confidence interval; HDL-c: high-density lipoprotein; HOMA-IR: homeostatic model assessment - insulin resistance; hs-CRP: ultrasensitive c-reactive protein; LDL-c: low-density lipoprotein; OR: odds ratio; TC: total cholesterol; Tg: triglycerides; VLDL-c: very high-density lipoprotein.

* Unadjusted model;

** Model adjusted for sex, age, years of education, and level of physical activity, considering individuals without obesity and without sarcopenia as a basis;

*** Statistically significant.

## Discussion

The implications of alterations in glycemic, lipidic, and inflammatory biomarkers on body composition phenotypes (sarcopenia, obesity, and SO) were explored. Using multinomial logistic regression models, we found that the obesity phenotype is significantly associated with increased blood glucose and changes in lipid biomarkers (TC, LDL-c, VLDL-c, non-HDL-c, Tg, and reduced HDL-c). SO was significantly and independently associated with insulin resistance, inflammation, and alteration of lipid biomarkers (LDL-c; not HDL-c).

We found a high prevalence of the obesity phenotype, which was significantly higher in females. Moreover, we noted that individuals with phenotypes of sarcopenia and SO (according to DXA data) had a mean BMI compatible with normal weight, according to the traditional classification, which indicates that these body composition phenotypes could not be accurately diagnosed by BMI.

### Characterization data

This study consisted of a younger population, more physically inactive, with greater parity in terms of sex in the whole sample, when compared to other studies that aimed to verify the prevalence of body composition phenotypes and sedentary profiles in the Brazilian population. In a study by Campos et al. ^25^, the participants had a mean age of 77.5 years, and in a study by Dutra et al. ^26^, the mean age was 66.6 years. Both values are higher than those found in our study. Furthermore, in the study by Campos et al. ^25^, most participants were female (70%) and, in the study by Dutra et al. ^26^, the entire sample was female, unlike this study that presented sex parity. Among Brazilian studies, the only one that verified whether individuals were physically active was that of Dutra et al. ^26^, in which 86% of the sample was considered physically active. However, in this study, only 21.7% of the sample was classified as physically active. Similar studies classifying sedentary profile were not found in the Brazilian population.

### Prevalence of body composition phenotypes

The prevalence of SO found in this study (6.17%) was very inferior to the results obtained by Campos et al. ^25^ and Dutra et al. ^26^, who found SO prevalence values of 29.3% and 20.8%. However, the studies by Campos et al. ^25^ and Dutra et al. ^26^ evaluated SO in older adults and our research evaluated young adult and adult individuals, so it was expected that we would find a lower prevalence, reaffirming the tendency of an increase in the prevalence of SO with advancing age. This expectation is based on previous findings that adults aged from 20 to 59 years have a prevalence of estimated SO ranging 5-10% ^27^. Moreover, Campos et al. ^25^ found a high prevalence of obesity in the sample (44.2%), especially in females (60.3%) when compared to males (7.4%). In our study, a high prevalence of obesity (62.5%) was also observed, which was statistically higher in females (70.5%) when compared to males (54.4%). Regarding the age group, when compared, individuals under the age of 60 years tend to have a higher prevalence of obesity without the presence of sarcopenia than those aged 60 years or over ^28^. These findings suggest that the adult Brazilian population has a higher prevalence of obesity than the older population, especially in women, with the possibility of developing SO in the future, considering that aging is a key factor in reducing the amount of muscle mass and that this process can be favored by excess adiposity ^28^. Furthermore, a statistically significant difference was observed in the prevalence of SO between sexes, which was higher in females, corroborating the findings by Kim et al. ^5^ and opposing those by Campos et al. ^25^. The result obtained agrees with the physiologically expected, as female individuals tend to have more adipose tissue mass and less muscle mass when compared to males ^29^.

### Sarcopenic obesity, body composition and BMI

The mean BMI of the SO group was similar to the group without sarcopenia and obesity. However, the mean percentage of body fat was higher in the group with SO phenotype when compared to individuals without sarcopenia and obesity, which demonstrates that even with an exacerbated increase in fat percentage, individuals with SO do not show change in the mean value of BMI. This may be due to the lower amount of muscle mass (kg) observed, as the SO group had the lowest muscle mass value, with a statistically significant difference when compared to the other groups, which in turn resulted in a BMI compatible with a normal weight diagnosis (according to the World Health Organization - WHO ^16^) but with major changes in body composition (verified by DXA). This may be one of the causes responsible for the underreporting of SO worldwide. For instance, a recent systematic review found that 23 out of 66 studies used BMI as the standard for diagnosing obesity, in the context of SO ^8^. In clinical practice, usually only BMI is used and the body composition is not assessed, which can result in patients with unidentified SO. This supports the hypothesis that the verification of body composition is the most recommended for the diagnosis of SO ^15^. Despite this, in clinical practice, the assessment of body composition by DXA is not accessible and, therefore, it is necessary for body composition to be defined by other accurate means, such as skinfolds. Moreover, despite being present, it is possible that obesity (defined by the percentage of fat) is not as noticeable in individuals with SO. This suggests that estimating the percentage of fat and quantifying the muscle mass of individuals while they are young and/or adults can be an effective way of prevention, allowing professionals to intervene before SO development.

### Association between body composition phenotypes and glycemic biomarkers and hs-CRP

We verified that increased blood glucose was associated with a greater odd of presenting the phenotypes of sarcopenia and obesity. Otherwise, there was no association between increased blood glucose and the odds of having SO phenotype. However, regarding HOMA-IR, the most recommended marker to verify IR ^30^, we observed that increased values resulted in 430% odds of presenting the obesity phenotype and 294% of presenting SO. The same result was observed in a study by Kim et al. ^5^, in which SO was independently and significantly associated with IR in both sexes. These findings raise the concern that individuals with IR and installed obesity can be at greater risk of developing SO in the future since IR can promote skeletal muscle catabolism ^4^ and IR tends to increase with aging. This may result in glycemic disorders such as pre-diabetes mellitus or type 2 diabetes mellitus ^26^.

Inflammation is an important mediator of IR ^31^ and pro-inflammatory cytokines can be critical in both development and progression of SO ^4^. Our results indicate that elevated hs-CRP is associated with a 152% increase in the individual’s chance of being obese and 142% of having the SO phenotype, which suggests that the inflammatory biomarker hs-CRP could be used as a predictor of SO. Furthermore, individuals with increased hs-CRP and installed obesity may be at greater risk of developing SO in the future, with increased risks when these factors coexist with IR.

When comparing individuals with and without SO and, in a study by Dutra et al. ^25^, mean values ​​of hs-CRP were found to be higher in the SO group, however, the difference was not significant. This may have occurred due to the sample size of the study, which was 130 participants, 27 in the SO group and 103 in the group without SO. In this study, a significant increase in the chance of an individual with high hs-CRP to have SO phenotype was verified. Our findings agree with the results of another cross-sectional study that evaluated the association between SO and hs-CRP and identified that SO is independently associated with inflammation in females ^6^.

### Association between body composition phenotypes and lipid biomarkers

LDL-c and non-HDL-c biomarkers showed a positive and significant association with both obesity and SO phenotypes. TC, VLDL-c, HDL-c, and Tg were associated with obesity, especially HDL-c, showing a negative association in which every 1mg/dL of HDL-c decreased in 3% the odds of the individual being obese. Participants with increased TC and those with increased triglycerides were associated with an increased chance of presenting the obesity phenotype. Our findings lead us to believe that higher levels of lipid biomarkers, especially LDL-c and non-HDL-c, show greater impact in obesity than in sarcopenia. In a study by Habib et al. ^32^, TC, Tg, and HDL-c were shown to be significantly related to SO in males, when compared to individuals without SO, suggesting that sarcopenia can exacerbate the clinical setting of dyslipidemia. Our study also points to this result, but with emphasis on LDL-c and non-HDL-c in both sexes. A cohort study conducted with individuals aged 50 years or more also found an association between LDL-c and SO, as well as TC, Tg, LDL-c, and HDL-c in individuals diagnosed with obesity ^33^.

### Strengths and limitations

As limitation of the study, we highlight that both dependent and independent variables were measured at the same time, which means we cannot guarantee that the exposure variables preceded the outcomes. Since this is a cross-sectional study, we verified the association between the dependent and independent variables and not the relationship of cause and consequence ^34^. Other limitation was that we did not consider the loss of muscle function or strength (dynapenia) ^7^. However, previous studies also defined sarcopenia as a reduction in muscle mass ^8^, which allows more reliable comparisons between the results of this study and others. As a strength, the assessment of muscle mass was performed using a standardized technique and DXA, considered the gold standard method for verifying body composition, which increased and ensured the accuracy of definition.

The study’s strongest point is the population-based design, with a representative sample of the population, which allows inferences to be made. Furthermore, we highlight that we found no studies evaluating the association between body composition phenotypes and glycemic, lipidic, and inflammatory biomarkers in the adult Brazilian population. We also emphasize the methodological rigor used in conducting the study, which included stages of training, calibration, pilot study, and quality control of data reproducibility, as previously described ^9^.

In conclusion, our results provide evidence that the SO phenotype is significantly and independently associated with IR, inflammation, and altered lipid biomarkers, even after adjusting for confounding factors. Our findings indicate that increased hs-CRP, HOMA-IR, LDL-c, and non-HDL-c appear to be important indicators of the SO phenotype and that increased glycemia, TC, Tg, LDL-c, VLDL-c, non-HDL-c, and decreased HDL-c may be indicators of the obesity phenotype. These parameters may be used as additional markers for screening obesity, sarcopenia, and SO phenotypes. Finally, this study reinforces the importance of new interventions aiming the improvement of these parameters in the young and adult population, preventing progression and/or installation of SO and other metabolic disturbances.
